# Treatment with l-citrulline and metformin in Duchenne muscular dystrophy: study protocol for a single-centre, randomised, placebo-controlled trial

**DOI:** 10.1186/s13063-016-1503-1

**Published:** 2016-08-03

**Authors:** Patricia Hafner, Ulrike Bonati, Daniela Rubino, Vanya Gocheva, Thomas Zumbrunn, Nuri Gueven, Dirk Fischer

**Affiliations:** 1 Division of Neuropaediatrics, University of Basel Children’s Hospital, Basel, Switzerland; 2 Division of Neurology, Medical University Clinic, Kantonsspital Baselland, Bruderholz, Switzerland; 3 Department of Clinical research, Clinical Trial Unit, University of Basel Hospital, Basel, Switzerland; 4 Pharmacy, School of Medicine, University of Tasmania, Hobart, TAS Australia; 5 Department of Neurology, University of Basel Hospital, Basel, Switzerland; 6 Division of Neuropaediatrics, University Children’s Hospital, Spitalstrasse 33, Postfach Basel, 4056 Switzerland

**Keywords:** l-citrulline, Metformin, DMD, Clinical trial, Quantitative MRI, Mitochondrial function

## Abstract

**Background:**

Duchenne muscular dystrophy (DMD) is an X-linked recessive disease that affects 1 in 3500–6000 male births. Despite broad research aiming to improve muscle function as well as heart and brain function, sufficient therapeutic efficacy has not yet been achieved and current therapeutic management is still supportive. In a recent pilot trial, oral treatment with l-arginine and metformin showed consistent changes of muscular metabolism both in vitro and in vivo by raising NO levels and expression of mitochondrial proteins in the skeletal muscle tissue of patients with DMD. This randomised, double-blind, placebo-controlled trial aims to demonstrate the superiority of l-citrulline and metformin therapy over placebo in DMD patients with regard to the Motor Function Measure (MFM) D1 subscore (primary endpoint) as well as additional clinical and subclinical tests.

**Methods/Design:**

A total of 40–50 ambulant patients with DMD will be recruited at the outpatient department of the University of Basel Children’s Hospital (Switzerland), as well as from the DMD patient registries of Switzerland, Germany and Austria. Patients will be randomly allocated to one of the two arms of the study and will receive either a combination of l-citrulline and metformin or placebo for 26 weeks. Co-medication with glucocorticoids is allowed. The primary endpoint is the change of the MFM D1 subscore from baseline to week 26 under l-citrulline and metformin therapy. Secondary endpoints will include the motor function measure (MFM) and its items and subscores, the 6-minute walking test, timed function tests and quantitative muscle testing. Furthermore, quantitative muscle MRI assessment to evaluate the muscle fat fraction as well as safety and biomarker laboratory analyses from blood will be included. For comparison, muscle metabolism and mitochondrial function will be analysed in 10–20 healthy age-matched male children.

**Discussion:**

The aim of this study is to test if a 6-month treatment of a combination of l-citrulline and metformin is more effective than placebo in preventing loss of motor function and muscle degeneration in DMD. The MFM D1 subscore is used as a clinical outcome measure and a quantitative muscle MRI assessment as the surrogate outcome measure of fatty muscle degeneration.

**Trial registration:**

ClinicalTrials.gov: NCT01995032. Registered on 20 November 2013.

**Electronic supplementary material:**

The online version of this article (doi:10.1186/s13063-016-1503-1) contains supplementary material, which is available to authorized users.

## Background

Duchenne muscular dystrophy (DMD) is characterized by rapid and irreversible replacement of normal muscle through connective and adipose tissue. The disease-causing gene product, dystrophin, is present in many different tissues throughout the body; however, the disease pathology is predominantly seen in skeletal and heart muscle. Altered neuronal nitric oxide synthase (nNOS), mitochondrial dysfunction and increased intracellular calcium (Ca^2+^) concentrations have previously been described as impairing cellular function in patients with DMD [1–7]. NO stimulates mitochondrial biogenesis by increasing sirtuin 1 (SIRT1) and peroxisome proliferator-activated receptor-γ coactivator-1α (PGC-1α) concentrations [8] and is also critical for regulating muscle energy balance by activating AMP-activated protein kinase (AMPK) [9]. NO and AMPK may synergistically increase mitochondrial function and biogenesis through independent mechanisms. Therefore, impaired nNOS function likely contributes to the observed mitochondrial dysfunction in DMD. Children with DMD show an elevated synthesis of asymmetric dimethylarginine (ADMA), a diminished homoarginine (hArg) synthesis and a reduced NO bioavailability compared to healthy children [10]. To increase NO levels to stimulate mitochondrial function, reduce oxidative stress and improve fat utilisation for energy production appears to be a promising approach to ameliorate the disturbed muscle cell metabolism in DMD. Activation of nNOS in skeletal muscle is AMPK dependent [11], and there is significant evidence for a beneficial effect of AMPK activation in the *mdx* mouse model of DMD. Chronic AMPK stimulation triggers beneficial adaptations [12] and ameliorates the dystrophic phenotype in the *mdx* mouse model [13]. One of the best-known pharmacological AMPK activators elevating AMPK concentrations in human skeletal muscle is metformin [14]. Consistent with its described activation of AMPK, metformin stimulates PGC-1α expression in the *mdx* mouse [15] and protects skeletal muscle from degeneration [16]. In summary, there is evidence that metformin in its role as AMPK activator substitutes deficient nNOS function in patients with DMD. This is achieved by direct stimulation of nNOS and independent increase in PGC-1α expression, both stimulating mitochondrial biogenesis.

To test the hypothesis of a synergistic effect of NO and AMPK to stimulate mitochondrial function, we conducted a proof-of-concept pilot trial in five patients with DMD aged between 7 and 10 years [17]. That study evaluated the subclinical and clinical benefits of a combined therapy with the NO precursor l-arginine, and the pharmacological AMPK activator and indirect nNOS stimulator metformin over a treatment period of 16 weeks. The results of this trial were encouraging, as the treatment in mostly steroid-naïve patients showed increased NO formation in DMD muscle as expected. More importantly, we also observed a positive correlation between increased markers of NO concentration and mitochondrial protein expression, indicating a direct relation between intramuscular NO and mitochondrial protein expression in DMD muscle. Motor function measure (MFM) scores and walking distances improved, whereas ambulatory DMD patients older than 7 years usually show a high annual decrease of MFM total and D1 subscores [18]. Despite the small number of patients, the MFM total score (+3.6 %) and the D1 subscore (+6.2 %) improved in comparison to published natural history data and data for the standard treatment with steroids. Untreated ambulatory DMD patients older than 7 years showed a median annual total MFM decrease of –7.9 % (D1 subscore –17.2 %) [19]. Additional evidence for a disease-slowing effect of the combination treatment was provided by thigh muscle magnetic resonance imaging (MRI) data, which did not show any significant increase of the thigh muscle or body fat fractions during the time of the study as expected, based on natural history data. This pilot trial could confirm the theoretical concept of an amelioration of muscular metabolism by stimulating mitochondrial function. However, the number of patients was too small to generate significant clinical evidence for a clear therapeutic effect. Thus, we will conduct a double-blind, randomised, placebo-controlled efficacy and safety study to prove efficacy of the treatment using clinical, laboratory and imaging outcome measures as endpoints.

Recently, it was shown that not only l-arginine, but also l-citrulline, increases nitric oxide generation in humans. Furthermore, the intake of l-citrulline leads to higher peak concentrations of l-arginine than an equivalent dose of l-arginine itself [20]. l-citrulline is a precursor of l-arginine; three-fourths of l-citrulline are converted to l-arginine in the kidney [21]. Single oral doses up to 15 g have been well tolerated without side effects [22]. Supplementation of l-citrulline restores muscular l-arginine and reduces muscle wasting under l-arginine-deficient and low-protein intake conditions [23, 24]. Additionally, a direct protective effect of l-citrulline on protein metabolism and skeletal muscle mediated through the inducible NOS (iNOS) isoform has been suggested [25]. These considerations led us to change administration of oral l-arginine to l-citrulline instead.

## Methods/Design

### Study design

The study is an investigator-initiated, double-blind, randomised, placebo-controlled efficacy and safety trial and is conducted over a treatment period of 26 weeks. We plan to enroll 40–50 ambulant patients with DMD aged between 6.5 and 10 years. Ethical approval has been obtained from the local ethics committee (Ethics Committee of Both Basel cantons (EKBB EK63/13)) and the National Swiss Drug Agency (Swissmedic). The trial was registered at ClinicalTrials.gov (NCT01995032) prior to starting recruitment. Patients and parents are being informed about preclinical data, alternative treatments, risks and possible benefits of the study. Oral informed assent from affected children and written informed consent from parents are being obtained. The participation in this study is voluntary. If the patient or the parents do not want to participate, they will not experience any disadvantages concerning further medical treatment. The same applies if parents and patients withdraw their consent at a later time. Patients or caregivers can withdraw their consent or withdraw from the study without giving any reason. In case of withdrawal, the data collected until this point of time will be used, and the (blood) samples collected in the context of the study will be destroyed. In case of withdrawal, the patient will undergo a final visit for medical examination for his own safety. To obtain normal values for measures of muscle metabolism and mitochondrial function, blood and urine samples are analysed in 10–20 healthy age-matched male children. Data is collected at the University Children’s Hospital in Basel, Switzerland.

### Inclusion criteria

Inclusion criteria are molecular diagnosis of DMD; 6.5–10 years of age at time of screening; ambulant patients with ability to walk 150 m in the 6-minute walking test (6MWT); D1 subdomain of the MFM scale >40 %; stable treatment with steroids for >6 months or steroid-naïve patients.

### Exclusion criteria

Excluded are patients with a previous (within the past 3 months) or concomitant participation in any other therapeutic trial; the use of l-citrulline, l-arginine or metformin within the last 3 months; a known individual hypersensitivity to l-citrulline or metformin; a known or suspected malignancy; any other chronic disease or clinical relevant limitation of renal, liver or heart function according to the discretion of the investigator; or the start of glucocorticoid treatment or change in dosage <6 months prior to screening.

### Randomisation and blinding

Patients that meet the study admission criteria are enrolled in the study and are assigned a single subject identification number. Patients are subsequently allocated to the two study groups, l-citrulline and metformin or placebo, according to a mixed randomisation scheme with an unbalanced starting block of 5 patients and subsequent randomly permuted balanced blocks of 2 or 4 patients [26]. The trial is double-blind, with both participants/parents as well as investigators assessing outcomes blinded to treatment allocation. The patients randomised to the placebo group receive a matching placebo with sachets and powder as well as capsules looking like verum but not containing active ingredients. Drop-outs after the baseline visit are not replaced.

### Intervention

**Fig. 1:**
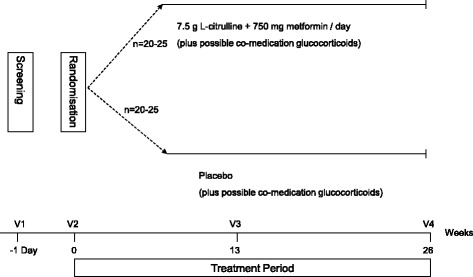
Flow chart showing the study design

All patients randomised to the active compounds receive l-citrulline (Hospital pharmacy, Basel, Switzerland) 2.5 g/d t.i.d. as well as metformin (metformin hydrochloride, Hospital pharmacy, Basel, Switzerland) at a dose of 250 mg t.i.d. Patients allocated to the placebo group receive matching placebo (mannitol) with sachets and powder as well as capsules looking like verum (l-citrulline sachets and metformin capsules). Treatment is given for a period of 26 weeks. Co-medication with glucocorticoids (standard treatment) is allowed and continued if treatment was started >6 months before randomisation. At baseline as well as at the end of the study, clinical measures, laboratory and MRI measures are performed. This includes the MFM scale, 6MWT, MFC assessment using quantitative thigh muscle MRI and laboratory blood analysis (Fig. 1).

### Study procedure

At screening (visit 1, day –1), patients and parents are informed about preclinical data, alternative treatments, risks and possible benefits of the study. Further, oral informed assent from affected children and written informed consent from parents are obtained. After signing the informed consent form, the inclusion and exclusion criteria are verified. If the criteria are fulfilled, the patient will be enrolled in the study. During this screening visit the following procedures are performed: vital signs, physical examination, MFM scale, 6MWT, muscle force of knee extension and elbow flexion using hand-held dynamometry and 10 m within 10 s walking test. After screening, visits will be scheduled for 0 (visit 2, baseline), 13 (visit 3) and 26 (visit 4, end of study) weeks.

During visit 2 (week 0, baseline) the following procedures are performed: check inclusion/exclusion criteria, adverse events and vital signs, physical examination, blood draw (laboratory analysis) and muscle MRI. If the patient still qualifies for the study, he will be randomised and receive the corresponding study medication. During visit 3 (week 13) the following procedures are performed: check for adverse events, dispensing of study medication, collection of bottles and boxes with sachets of used study medication for compliance control, check vital signs, physical examination, blood draw (laboratory analysis), MFM scale, 6MWT, muscle force of knee extension and elbow flexion using hand-held dynamometry and a 10 m within 10 s walking test.

During the last visit (visit 4, week 26, end of study) the following procedures are performed: check for adverse events, collection of study medication, check vital signs, physical examination, blood draw (laboratory analysis), MFM scale, 6MWT, muscle force of knee extension and elbow flexion using hand-held dynamometry, 10 m within 10 s walking test and muscle MRI (Table 1).

**Table 1:** Patient timelines / scheme of interventions

Visit	1	2	3	4
Week	Day -1	0	13	26
Enrolment:				
Informed consent	X			
Inclusion/Exclusion	X	X		
Randomisation		X		
Interventions:				
Treatment				
Placebo				
Dispensing of study medication		X	X	
Collection of study medication			X	X
Metformin serum concentration (compliance check)				X
Assessments:				
Medical history and demographics	X			
Capture Adverse Events		X	X	X
Vital Signs (blood pressure, heart rate, weight, height	X	X	X	X
Physical examination (general and neurological)	X	X	X	X
Blood and urine analysis: amino acids, haematology^b^, chemistry^c^, markers of oxidative stress and NO function		X	X	X
MFM scale	X^a^		X	X
Muscle force of knee extension and elbow flexion using hand held dynamometry	X^a^		X	X
6MWT	X^a^		X	X
Timed function test: 10 m walking test, Supine up	X^a^		X	X
MRI		X		X

^a^Will be used as baseline values

^b^Full blood count: erythrocytes, reticulocytes, leucocytes, platelets, haemoglobin, haematocrit

^c^Liver and renal function tests, electrolytes, urea, creatine kinase, HbA1c, cholesterol, high and low density lipoprotein (HDL, LDL) triglycerides

### Healthy controls

Between 10 and 20 healthy age-matched boys are enrolled to obtain normal values of mitochondrial and muscle metabolism. Healthy children and their caregivers are asked to participate in our study while undergoing an elective surgery at our hospital. Blood is collected as part of the routine safety protocol while the child is under anaesthesia. An additional amount of 4 ml of blood is drawn, but no additional venous puncture is necessary for this study. At screening (visit 1, day –1) patients and parents are informed about the procedure. Oral informed assent from the children and written informed consent from parents are obtained. If the inclusion criteria are fulfilled and no exclusion criteria are met, the patient will be enrolled in the study. During the baseline visit the inclusion and exclusion criteria are checked and a urine sample is taken. Blood collection is performed the same day, as explained above.

#### Inclusion criteria healthy volunteers

The inclusion criteria are male sex and aged between 6.5 and 10 years.

#### Exclusion criteria healthy volunteers

The exclusion criteria are previous (within the past 3 months) or concomitant participation in any other therapeutic trial, use of l-citrulline, l-arginine or metformin within the last 3 months, known genetic or acquired neuromuscular disorder, known or suspected malignancy and other chronic disease or clinical relevant limitation of renal, liver or heart function according to the discretion of the investigator.

### Quality assurance

To assess high-quality conduct of the trial in accordance with the protocol, all medical staff involved in this study are certified in good clinical practice (GCP). Moreover, physiotherapists are certified to perform the motor function measures. To assess compliance of medication intake, empty and full bottles and boxes with sachets are returned by the patients at visits 3 and 4.

### Safety assessments

Adverse events are monitored throughout the study. At every study visit patients and caregivers are asked about adverse events, a clinical examination is performed and the vital parameters are measured. The following safety parameters amongst other parameters are checked: a full blood count and clinical chemistry (transaminases, creatinine, electrolytes, urea) will be measured as well as creatine kinase concentration as a marker of muscle necrosis. The intake of metformin will be stopped in case of an increase of creatinine and transaminases >2 × upper limit of baseline. Withdrawal of consent, protocol violations caused by the patients (noncompliance), logistical reasons, inability to attend study visits as defined in the protocol or abnormal laboratory results, including liver or renal function tests and abnormal increase in blood pressure as determined by the investigator can lead to an early termination of the study.

Patients with adverse reactions which have occurred during the study are followed up by the investigator up to 30 days after the last visit.

If pathologic changes independent of the known muscle disease are detected, the affected patients will be informed immediately, and the need for further diagnosis and treatment according to current medical knowledge will be discussed.

### Outcome measures

#### Primary outcome measure

##### Change of MFM D1 subscore from baseline to week 26 under l-citrulline and metformin therapy

Several tests have been reported in the literature to assess muscle strength and functional ability, to monitor the progression of the disease and to evaluate the results of drug interventions and rehabilitation. However, most instruments can assess ambulant patients only, making adjustments and/or additional assessments necessary when the disease progresses. The MFM, a validated assessment tool to measure motor function in both ambulant and non-ambulant patients with neuromuscular disorders, was developed in 2005 in France. It includes 32 items that evaluate three dimensions of motor performance, including specific motor functions, such as transfers and standing posture (D1), proximal and axial (D2), distal (D3) and a total MFM score involving all of the motor dimensions. The items are scored and summarised to comprise a total score, in which the maximum score represents normal motor function. The instruction manual, validation examinations and other publications using the MFM can be downloaded at the MFM website (http://www.motor-function-measure.org/home.aspx). In a recent study, the annual decrease of the total score was 5.8 % for patients with DMD, indicating an overall decline in motor capacities [27]. However, independent examination of the D1, D2 and D3 subscores provided more information depending on the stage of the disease. In ambulant DMD patients, D1 was the most informative score, with a mean decrease of 17.2 % per year before loss of ambulation in patients aged 6 years and older. D1 seems to us to be particularly interesting because it is related to loss of ambulation and is responsive to short-term changes (3 months) and should provide sufficient information in a 6-month trial. Consistent with this, in our previous 16-week l-arginine and metformin pilot trial in ambulant patients with DMD, the D1 subscore provided the best effect size of all evaluated clinical parameters [17].

#### Secondary outcome measures

##### Change of MFM total score, the D2 and D3 MFM subscores from baseline to week 26 under l-citrulline/metformin therapy

According to Bushby et al. [27], D2 is the most informative subscore after loss of ambulation, with an average decrease of 9.4 % per year. In patients older than 14 years, the average decrease in D3 was 10.8 % per year [27].

##### Change of walking distance assessed with the 6-minute walking test

Timed clinical function tests, like the 6-minute walking test (6MWT), are helpful in assessing muscle function in children with DMD. The 6MWT has been validated for use in DMD trials and measures the distance an individual is able to walk over a total of 6 minutes on a hard, flat surface. The goal for the person is to walk as far as possible in 6 minutes. The individual is allowed to self-pace and rest as needed as they traverse back and forth along a marked walkway. Data from 112 DMD patients aged 4–17 years in an Italian cohort showed that the 6MWT performances increased (approximately 33 m/year) with age up to 7 years, with a clear point of slope change at approximately 7 years. After the age of 7 years, there was a variable decline in the 6MWT (–12 m/year) [28].

##### Change of quantitative muscle MRI including muscle fat fraction and T2 times of thigh muscles visualised by MRI

In muscular dystrophies quantitative muscle MRI (qMRI) detects disease progression more sensitively compared to other clinical scores. Excellent reproducibility of qMRI was demonstrated in healthy volunteers as well as in patients [29, 30]. The qMRI test was more sensitive than clinical evaluation and visual analysis of MRI scans to detect disease progression [31, 32]. As recently investigated in 20 DMD patients, muscle fat fraction (MFF) in patients with DMD shows excellent correlation to clinical parameters, but is less variable and is therefore useful as a surrogate outcome parameter in patients with DMD [33]. Sample size estimations for qMRI data are up to 17 times smaller compared to the MFM total score and up to 7 times smaller compared to the D1 subscore, respectively.

##### Change in plasma and urine laboratory parameters

Aims of our treatment are: (1) an elevation of NO concentrations measured by indirect markers such as nitrotyrosine, nitrate, nitrite, asymmetric dimethylarginine (ADMA)); (2) an improvement of muscular energy state with reduction of oxidative stress measurable by 8-hydroxydeoxyguanosine (8OHDG) in urine and carbonylated protein concentration in the serum; (3) a slowing of muscle degeneration measured by creatine kinase, transaminases and alkaline phosphatase levels in serum.

##### Normal values for markers of oxidative and nitrosative stress and miRNA in serum and urine

Normal values of indirect markers of NO concentrations as well as oxidative stress will be measured in a healthy cohort of 10–20 male children aged 6.5 to 10 years.

##### Change of quantitative muscle testing of knee extension and elbow flexion using hand-held dynamometry

To measure muscle force independent of muscle function and aspect, quantitative muscle testing (QMT) of knee extension and elbow flexion will be done using a dynamometer [34].

### Randomisation scheme

Since a complete blinding of the physicians involved in the trial cannot be guaranteed, a mixed randomisation scheme is used following the recommendation of Schulz and Grimes [26]. An unequal starting block of size 5 is generated using replacement randomisation. Then randomly permuted blocks with equal numbers of assignments to the two groups (verum and placebo) are appended. Block sizes are 2 or 4 and are selected randomly with equal probability. For emergency breaking of the group assignment, unblinding envelopes are provided. These envelopes have a window in which only the running patient's ID is visible and contain an unblinding form that states the patient's group assignment.

### Sample size estimation

**Fig. 2:**
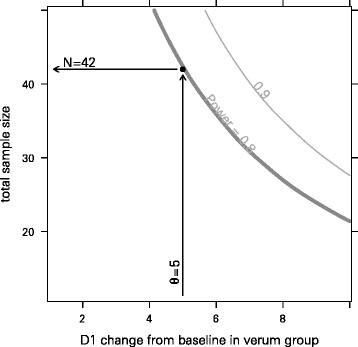
Sample size estimation. Sample size with regard to the MFM D1 subscore and the power for a randomisation ratio of 1:1. The numbers on the curves indicate the power. The *arrows* show how to interpret the plot. The curves are smoothed and only serve illustrative purposes

Sample size estimation was based on the primary endpoint MFM D1 subscore. A semi-parametric approach was chosen that made use of our own data from a comparable pilot trial [17]. Each sample size, *n*_*i*_ = _1,…,40_ = 11, …, 50, was evaluated by drawing 99 times an individual data set of size *n*_*i*_ from the pilot study data set (sampling with replacement). In each of these individual data sets, each patient was randomly assigned to the verum or a placebo group. For each patient, the post-treatment value was calculated as the sum of the baseline value and a random variate drawn from a normal distribution. Its standard deviation was estimated from the pilot data. For the placebo group, the mean change from baseline was set to –17.2/52 × 16 = –5.3 (value taken from Table 2 in [27]). (The value from [27] refers to changes within one year, i.e. approximately 52 weeks, and the pilot study lasted 16 weeks, thus the linear transformation of the values by /52 × 16.) For the verum group, the mean change from baseline was varied from 1 to 10 (in the pilot data, the mean change from baseline was 6.1). The resulting data set was analysed with an ANCOVA model (as outlined in the next section). The proportion of repetitions with a significant outcome corresponds to the estimated power. Assuming a drop-out rate of 10 %, a significance level of 0.05 and a power of 0.8, 47 patients should be recruited in order to be able to analyse 42 complete data sets. Figure 2 shows how the sample size behaves with regard to the MFM D1 subscore and the power.

### Statistical analyses

The hypothesis is that patients in the verum group will have a larger MFM D1 subscore than patients in the placebo arm after 13 or 26 weeks. If assumptions are not violated, ANCOVA models are used for the primary analysis. The response variable is the D1 subscore after 13 or 26 weeks; i.e. separate models for the 13 and 26 weeks follow-up measurements are formulated. The explanatory variables are D1 at baseline and trial group. The model estimates are presented together with 95 % confidence intervals and *p* values with respect to the null hypothesis that the corresponding coefficient is zero. In addition to the unadjusted analysis, an analysis adjusted by the binary variable indicating whether the patients were under cortisol treatment is conducted. The analyses are done following the intention-to-treat (ITT) principle. If assumptions of ANCOVA are violated, non-parametric ANCOVAs are used instead (Bowman and Azzalini [35]).

The analyses are repeated based on the per protocol (PP) principle. Deviations from the results of the ITT analysis are described in detail. Furthermore, the ITT analyses are repeated on an imputed data set where missing measurements at week 13 or 26 are set according to the last-observation-carried-forward (LOCF) principle. Deviations from the results of the non-imputed ITT analysis are described in detail. One subgroup analysis is performed based on patients’ baseline measurement of 6-minute walking distance, where subgroups are defined as walking distance >350 m or <350 m. The same procedure as used for the analysis of the primary endpoint is employed for the analysis of each subgroup. With the aim of generating hypotheses, the above procedure for the primary endpoint is applied to all secondary endpoints.

### Quality control

To assure the quality of the study conduct and of the data, monitoring of the study is performed by organisations independent of the study (Clinical Trial Unit, University Hospital Basel, Kammermann Monitoring Services GmbH). All inclusion and exclusion criteria are checked, and the monitor controls if the data have been recorded correctly in the case report form, if the drug accountability is correct and if serious adverse events have occurred during the study. A Standard Protocol Items: Recommendations for Interventional Trials (SPIRIT) checklist (Additional file 1) is added to the publication of the protocol.

## Discussion

We propose a single-centre, randomised, double-blind, placebo-controlled trial to determine whether treatment with l-citrulline and metformin has a positive effect on clinical function and paraclinical surrogate biomarkers in Duchenne muscular dystrophy (DMD). If the combined treatment of l-citrulline and metformin is proven to be effective, it will offer a new symptomatic treatment option to slow muscular decline and therefore extend the time of free ambulation.

Planning clinical trials in DMD is challenging for the following reasons. (1) Disease progression does not follow a linear course when physical abilities are measured. Longitudinal studies using clinical measurements showed an improvement of gross and fine motor function up to the age of 6 to 7 years, then a plateau phase up to the age of 10 and then a phase of rapid decline during which free ambulation is lost [18, 28]. (2) DMD is a rare disease, making recruitment for clinical trials difficult. (3) Clinical assessment is dependent on cooperation and often differs between ambulant and non-ambulant patients [33]. Several motor function or timed motor performance tests are validated to assess disease progression in DMD, such as the Northstar Ambulatory Assessment (NSAA) [28], the 6-minute walking distance [36] and the Hammersmith Functional Motor Scale [37]. The Motor Function Measure (MFM) is a validated clinical score recommended in the guidelines on the clinical investigation of medicinal products for the treatment of DMD published by the European Medicines Agency [38]. Its interrater variability is low [18]. It was shown to be highly correlated to age in patients with DMD and was even to some extent predictive for loss of ambulation in these patients. Loss of ambulation was shown to occur about one year after the MFM total score dropped below 70 %, or a drop in D1 subscore below 40 %, respectively [18]. A main advantage of the MFM over other commonly used motor function or timed motor performance tests is that it can be used continuously throughout the course of a disease regardless of the severity of the disease and the patient’s ambulatory status [38].

Recently, we demonstrated that out of all analysed clinical measures, the MFM D1 subscore showed the most significant decline in ambulant DMD patients >7 years, resulting in the highest effect size and the lowest required patient numbers to treat [33]. This score also seems to be particularly interesting because of its relation to loss of ambulation and its responsiveness to short-term changes. Therefore, to test our approach we selected the MFM D1 as the (primary) endpoint for the selected population of ambulant patients with DMD. For sample size estimation using the MFM D1 subscore as primary endpoint and assuming a drop-out rate of 10 %, a randomisation ratio of 1:1 and a power of 0.8, 42 patients should be recruited in order to be able to analyse 38 complete data sets. To be able to recruit sufficient patients in a reasonable time we choose to include patients with a walking distance of more than 150 m in 6 minutes. This is not optimal and is likely to include patients in a stable as well as a more unstable phase. Therefore, predefined subgroup analysis of patients walking more (stable group) or less (unstable group) than 350 m in the 6MWT has been planned.

To further increase the sensitivity to detect whether our intervention has a beneficial effect on muscle degeneration, we will use thigh muscle qMRI as a surrogate biomarker as the most important secondary endpoint. A surrogate (imaging) outcome measure must provide evidence of whether the treatment provides a clinically meaningful benefit. Therefore, it must (1) correlate with the clinical endpoint, (2) be sensitive to the effects of the intervention, (3) be clinically relevant to the disease and the treatment and (4) be readily measurable and interpretable [39]. The qMRI fulfils all these conditions, if protocols for qMRI are set up in advance with regard to patient positioning, slice selection and ROI delineation, and if the technical staff performing the examination and evaluation have been trained accordingly. Recently we demonstrated that in comparison to clinical endpoints the effect sizes of qMRI were much larger in DMD patients; e.g. sample size estimations for qMRI data were up to 17 times smaller compared to the MFM total score and up to 7 times for the D1 subscore, respectively [33]. Thus, in case our sample size estimation (using the clinical endpoint) was inaccurately low, qMRI should still be able to detect whether our intervention can affect disease progression positively.

The determination of normal values of markers of muscle metabolism and mitochondrial function in blood and urine in an age-matched healthy cohort will allow a better integration of an eventual stimulation of NO concentration and reduction of oxidative stress in treated patients with DMD.

### Trial status

The trial started enrolment in January 2014 and is expected to be completed by the end of May 2016.

## Abbreviations

6MWT, 6-minute walking test; 8OHDG, 8ydroxydeoxyguanosine; ADMA, asymmetric dimethylarginine; AMPK, AMP-activated protein kinase; Ca^2+^, calcium; DMD, Duchenne muscular dystrophy; hArg, homoarginine; MFF, muscle fat fraction; MFM, motor function measure; nNOS, neuronal nitric oxide synthase; NO, nitric oxide; PGC-1α, peroxisome proliferator-activated receptor-γ coactivator-1α; qMRI, quantitative muscle MRI; QMT, quantitative muscle testing; SIRT1, sirtuin 1; TFT, timed function tests

## Additional file

Additional file 1: SPIRIT checklist. (PDF 60.4 kb)
